# Countering TRAIL Resistance in Melanoma

**DOI:** 10.3390/cancers11050656

**Published:** 2019-05-11

**Authors:** Jürgen Eberle

**Affiliations:** Department of Dermatology, Venerology and Allergology, Skin Cancer Center Charité, Charité-Universitätsmedizin Berlin (University Medical Center Charité), 10117 Berlin, Germany; juergen.eberle@charite.de; Tel.: +49-30-450-518383

**Keywords:** melanoma, TRAIL, kinases, Bcl-2 proteins, Bax, Smac, XIAP

## Abstract

Melanoma of the skin has become a prime example for demonstrating the success of targeted cancer therapy. Nevertheless, high mortality has remained, mainly related to tumor heterogeneity and inducible therapy resistance. But the development of new therapeutic strategies and combinations has raised hope of finally defeating this deadly disease. TNF-related apoptosis-inducing ligand (TRAIL) represents a promising antitumor strategy. The principal sensitivity of melanoma cells for TRAIL was demonstrated in previous studies; however, inducible resistance appeared as a major problem. To address this issue, combination strategies were tested, and survival pathway inhibitors were shown to sensitize melanoma cells for TRAIL-induced apoptosis. Finally, cell cycle inhibition was identified as a common principle of TRAIL sensitization in melanoma cells. Mitochondrial apoptosis pathways, pro- and antiapoptotic Bcl-2 proteins as well as the rheostat consisted of Smac (Second mitochondria-derived activator of caspase) and XIAP (X-linked inhibitor of apoptosis protein) appeared to be of particular importance. Furthermore, the role of reactive oxygen species (ROS) was recognized in this setting. Inducible TRAIL resistance in melanoma can be explained by (i) high levels of antiapoptotic Bcl-2 proteins, (ii) high levels of XIAP, and (iii) suppressed Bax activity. These hurdles have to be overcome to enable the use of TRAIL in melanoma therapy. Several strategies appear as particularly promising, including new TRAIL receptor agonists, Smac and BH3 mimetics, as well as selective kinase inhibitors.

## 1. Still High Mortality of Melanoma Despite Efficient New Therapies (Introduction)

While the incidence of most solid tumors has decreased or at least stabilized in the last decades, the incidence of skin cancer has continued to rise worldwide [1]. It is particularly a problem of the Caucasian populations with light skin and too much UV radiation. Skin cancer is subdivided into melanoma and non-melanoma skin cancer, the latter mainly enclosing basal cell carcinoma and cutaneous squamous cell carcinoma. Other non-melanoma skin cancers such as Merkel cell carcinoma and cutaneous T-cell lymphoma are less frequent. In the United States, non-melanoma skin cancer is the most common and melanoma the sixth most common cancer [2]. The situation appears as comparable in Europe and even worse in Australia [3,4].

With regard to melanoma, the problems of early dissemination and pronounced chemotherapy resistance remained completely unsolved for decades [5]. Just in recent years, the situation significantly improved due to (i) the development of targeted therapy based on selective inhibitors for the MAP kinases BRAF (B-Raf proto-oncogene) and MEK (MAPK/ERK kinase) and (ii) due to the development of efficient immune-stimulating antibodies such anti-CTLA4 (cytotoxic T-lymphocyte-associated protein), anti-PD1 (programmed cell death), and anti-PDL1 (PD1 ligand). Together, these new strategies now enable a significant prolongation of overall survival of metastatic melanoma patients [6,7]. Nevertheless, for many patients, tumor relapse and therapy resistance often follow within only a few months or years, after a phase of initial tumor reduction [2,8]. Thus, new combination partners are still needed, which may further improve the clinical outcome. Many drug candidates have been investigated, and TRAIL (Tumor Necrosis Factor α-related apoptosis-inducing ligand) appears as promising.

## 2. Apoptosis Deficiency Is a Major Cause of Melanoma Therapy Resistance

A number of cellular mechanisms contribute to the development of cancer, which have been listed in the often-cited hallmarks of cancer [9]. Concerning therapy resistance, apoptosis deficiency may have the most decisive contribution. This is suggested by the principal need to finally eliminate the tumor cells, and apoptosis induction appears as the most common and most efficient way of doing so. Apoptosis is also the end path of many anticancer therapies in melanoma. Thus, chemotherapeutic drugs cause DNA defects or other kinds of cellular damage, which activate intrinsic, proapoptotic pathways in melanoma cells [10]. Also, BRAF inhibition in melanoma cells has been related to an induction of apoptosis as well as to a sensitization for other proapoptotic effectors. Thus, TRAIL-induced apoptosis was strongly enhanced, and TRAIL resistance was overcome in melanoma cells by BRAF inhibitors, including vemurafenib approved for melanoma therapy [11,12,13]. Finally, the stimulation of an anti-tumor immune response results in activation of cytotoxic T-lymphocytes, which also express death ligands to trigger extrinsic proapoptotic pathways in target cancer cells [14]. Therapeutic strategies that aim at the reinforcement of apoptosis pathways thus appear as important. Furthermore, sensitization of melanoma cells for TRAIL-induced apoptosis may support an anti-tumor immune response, also based on the expression of death ligands. In the light of the breakthrough of approved immune-stimulating therapies in melanoma, this issue gets a particular meaning [6,7].

## 3. Induction of Apoptosis by Death Ligands

**Figure 1:**
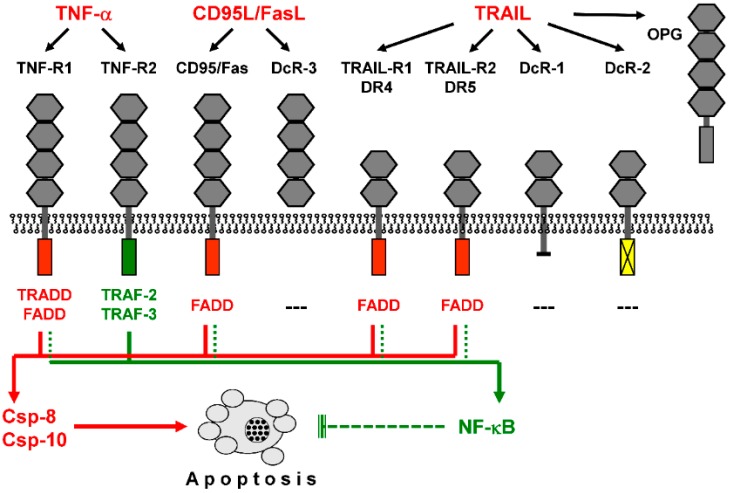
Death receptors activate proapoptotic caspase cascades as well as NF-κB. Binding of death ligands (TNF-α, CD95L/FasL, and TRAIL) to cognate receptors is shown. These share up to four cysteine-rich extracellular domains (grey hexagons). Death receptors (TNF-R1, CD95/Fas, TRAIL-R1/DR4, and TRAIL-R2/DR5) are further characterized by an intracellular death domain (red box), which allows binding of the adaptor protein FADD (Fas-associated death domain). TNF-R1 binds adaptor protein TRADD (TNF receptor-associated death domain), which further mediates FADD binding, while the intracellular domain of TNF-R2 (blue box) binds to TRAF proteins (TNF receptor-associated factors). Decoy receptors are characterized by lacking an intracellular domain (DcR-1), by a non-functional intracellular domain (DcR-2, yellow box), or by lacking a functional transmembrane domain (DcR-3, OPG, osteoprotegerin). FADD mediates proapoptotic caspase activation via caspase-8 and/or caspase-10 (red arrowheads) and may also support NF-κB (nuclear factor kappaB) activation, with mainly antiapoptotic functions (green arrowheads). In contrast, TNF-R2 via TRAF2/3 does not support caspase activation but activates NF-κB.

**Figure 2:**
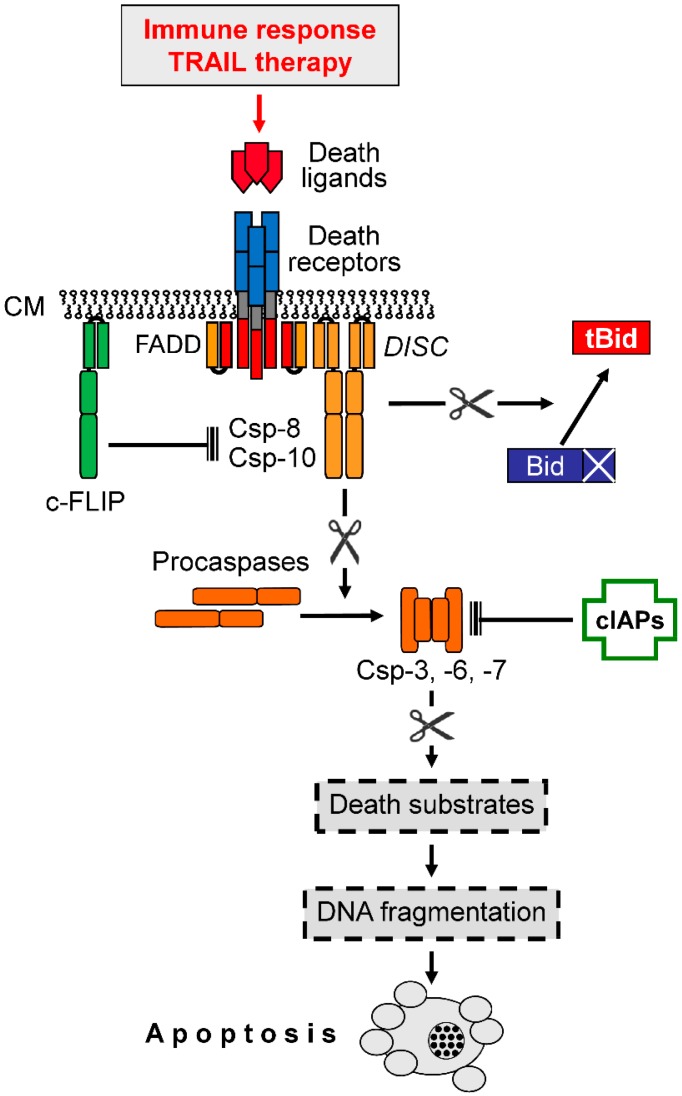
Extrinsic proapoptotic signaling. Binding of death ligands (e.g., TRAIL) to cognate death receptors (e.g., DR4, DR5) leads to the formation of a death-inducing signaling complex (DISC) within the cytoplasma membrane (CM), where initiator caspases-8 and -10 (Csp-8, Csp-10) bind via the adaptor protein FADD. Initiator caspases lead to the processing and activation of effector caspases (Csp-3, -6, -7), which mediate the processing of death substrates (point of no return). Csp-8, and -10 are negatively controlled by the competitive inhibitor, c-FLIP, while effector caspases are inhibited through binding of cellular inhibitor of apoptosis proteins (cIAPs). Caspase-8 can also activate a mitochondrial amplification loop via cleavage and activation of Bid to truncated Bid (tBid), a proapoptotic BH3-only protein. Arrowheads indicate activation while blunt ends indicate inhibition; scissors (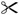) indicate protease activity.

Extrinsic proapoptotic pathways are triggered by death ligands such as TNF-α, CD95L/FasL, or TRAIL, which bind to cognate death receptors (TNF-R1, CD95/Fas, TRAIL-R1/DR4, and TRAIL-R2/DR5, Figure 1). Melanoma cells reveal principal sensitivity to CD95L as well as to TRAIL [15,16]. Death receptors can activate proapoptotic initiator caspases as well as the NF-κB pathway with mainly antiapoptotic functions [17]. In contrast, TNF-R2 is not linked to caspase activation, and decoy receptors work antagonistically, as they can bind death ligands but do not transduce the signal [18]. For TRAIL sensitivity of melanoma cells, decoy receptors (DcR1 and DcR2) may play a minor role, as only week expression was found in melanoma cell lines [19]. Ligand binding to death receptors leads to the formation of a death-inducing signaling protein complex (DISC, Figure 2), where initiator caspases-8 and -10 are activated by induced proximity and autocatalytic proteolysis [20,21]. Initiator caspases can drive the processing and activation of effector caspases-3, -6 and -7, which cleave a large number of so-called death substrates to irreversibly set apoptosis into work [22]. 

The extrinsic apoptosis pathway is negatively regulated by antagonistic proteins. Thus, c-FLIP (cellular FLICE-inhibitory protein) serves as a competitive inhibitor of caspase-8/-10; it can bind to the DISC but does not promote effector caspase activation [23,24]. Further downstream, the family of cellular inhibitor of apoptosis proteins (cIAPs) can bind to effector caspases and thus prevent their proteolytic activity [25,26] (Figure 2). Significant roles of both c-FLIP and XIAP (chromosome X-linked IAP) have been shown for TRAIL resistance of melanoma cells [27,28].

## 4. Induction of Apoptosis by Intrinsic Signals

**Figure 3:**
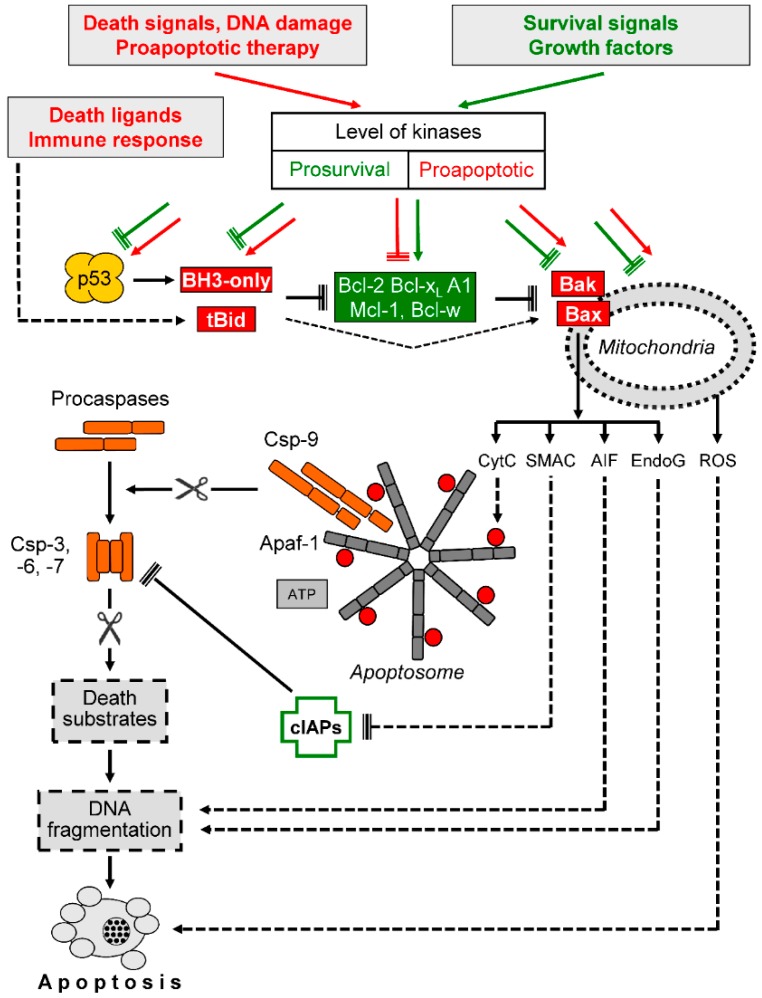
Intrinsic proapoptotic signaling. Death signals and survival signals affect the control level of cellular kinases in opposite directions (prosurvival and proapoptotic). Kinases may affect p53, proapoptotic BH3-only proteins, antiapoptotic Bcl-2 proteins (Bcl-2, Bcl-x_L,_ A1, Mcl-1, Bcl-w), proapoptotic, multidomain Bcl-2 proteins (Bax and Bak) as well as mitochondrial functions. The BH3-only protein Bid is cleaved by caspase-8 in response to death ligands, resulting in activated, truncated Bid (tBid). The activation of Bcl-2 proteins results in the release of proapoptotic, mitochondrial factors such as cytochrome c (CytC), second mitochondria-derived activator of caspase (Smac), apoptosis-inducing factor (AIF), and endonuclease G (EndoG). In contrast, reactive oxygen species (ROS) production may result from mitochondrial dysfunction but appears as largely independent of Bcl-2 proteins. Further abbreviations: Csp-3, -6, -7, -9, caspases; cIAPs, cellular inhibitor of apoptosis proteins; Apaf-1, apoptotic protease activating factor; ATP, adenosine triphosphate. Arrowheads indicate activation while blunt ends indicate inhibition; scissors (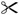) indicate protease activity. Proapoptotic factors and mechanisms are shown in red and orange, while antiapoptotic factors and mechanisms are shown in green. Further explanations are given in the text.

Intrinsic apoptosis pathways are initiated by different kinds of cellular dysregulation, e.g., by DNA damage and/or chemotherapy (Figure 3). A central event here is the proapoptotic activation of mitochondria, which encloses depolarization of the mitochondrial membrane potential (Δψm) and the release of proapoptotic mitochondrial factors, such as cytochrome c, second mitochondria-derived activator of caspases (Smac), apoptosis-inducing factor, and endonuclease G [29,30]. Cytosolic cytochrome c induces formation of the apoptosome, a multiprotein complex enclosing the adapter protein Apaf-1, which leads to initiator caspase-9 activation. Furthermore, Smac contributes to the activation of the caspase cascade, as it functions as an antagonist of cIAPs, e.g., of XIAP [31]. In contrast, apoptosis-inducing factor and endonuclease G contribute to apoptosis induction in caspase-independent ways, namely by supporting DNA fragmentation in the nucleus [32,33]. 

In many cells, extrinsic apoptosis pathways have to be enhanced through the mitochondrial pathway, which is based on processing and activation of the proapoptotic Bcl-2 protein Bid through caspase-8 [34,35]. In melanoma cells, direct caspase activation in response to death ligands (CD95L and TRAIL) appears as less active. Thus, the mitochondrial activation loop via Bid appears of particular importance, which is also shown by the critical role of Bcl-2 protein expression for death ligand sensitivity [15,27,36].

Mitochondrial permeability is critically controlled by Bcl-2 proteins, which enclose antiapoptotic (Bcl-2, Mcl-1, Bcl-x_L_, Bcl-w, and A1), proapoptotic multidomain (Bax and Bak), as well as a number of proapoptotic BH3-only proteins (e.g., Bid, Bim, Bad, Puma, and Noxa) [37]. Bcl-2 proteins are under tight self-control, based on their mutual heterodimerization (Figure 3). According to present models, mitochondrial permeability is mediated by the proapoptotic multidomain proteins Bax and Bak, which form mitochondrial pores or induce pore formation [38,39]. The antiapoptotic Bcl-2 proteins may heterodimerize with Bax or Bak to keep them in check. Different antiapoptotic Bcl-2 proteins may substitute each other [40,41], and in melanoma cells the particular roles of Bcl-2, Bcl-x_L,_ and Mcl-1 have been described [15,40,42]. 

The next control level is built up by the group of BH3-only proteins, which can heterodimerize with the different antiapoptotic Bcl-2 proteins in a competitive way to thus release Bax and Bak. BH3-only proteins can be activated in the course of various cellular stress situations either by induced expression, or by translocation and/or protein modification, e.g., phosphorylation [37,43]. In this way, BH3-only proteins function as sensitizers for apoptosis induction through inhibition of antiapoptotic Bcl-2 proteins. In addition, some of them may also directly activate Bax, as reported for Bid, Bim, and Puma [44]. Furthermore, several subsequent steps are regulated by cellular kinases, such as by phosphorylation of antiapoptotic Bcl-2 proteins, by phosphorylation of Bax, or by affecting mitochondrial functions to release ROS [11,45,46] (Figure 3). Due to the important role of intrinsic apoptosis pathways in melanoma cells, Bcl-2 proteins appear as critical targets for melanoma therapy [15,47], and particularly efficient apoptosis induction was found for Bim and Puma [38,48,49].

## 5. Critical Role of Apoptosis Deficiency in Melanoma

In normal tissue, homeostasis is maintained by a well-balanced equilibrium of cell proliferation and cell death [50]. Because of its critical meaning for the cell´s fate, apoptosis pathways are tightly regulated. Induction of apoptosis also serves as an important safeguard mechanism to prevent cancer by eliminating potentially harmful cells. A defective proapoptotic signaling thus represents a critical hallmark of cancer [9]. Apoptosis deficiency firstly permits initial tumor growth, and then it critically contributes to therapy resistance in advanced cancer. Apoptosis deficiency can be mediated by activation of antiapoptotic signals as well as by inactivation of proapoptotic pathways. Due to the important role of intrinsic, proapoptotic pathways in melanoma, pro- and antiapoptotic Bcl-2 proteins come into particular focus [47,51].

The pronounced chemotherapy resistance of melanoma is highly suggestive for deficient apoptosis programs [36]. Survival pathways are frequently activated, as seen by the high frequency of mutations in N-RAS (Rat sarcoma oncogene; 10–25%) and in BRAF (40–60%) [52]. The pathways of RAS/RAF/MEK/ERK and PI3K/AKT/mTOR appear as particularly promising targets for melanoma. Thus, selective BRAF inhibitors have been approved [53,54,55], and combinations of BRAF and MEK inhibitors as well as combinations of BRAF and PI3K/AKT inhibitors are presently evaluated [56,57,58]. The role of these pathways for apoptosis sensitivity of melanoma cells has been shown [13,59].

Activation of death receptors appears as an attractive and additional therapeutic strategy for cancer. Death ligands may induce apoptosis independently of p53, in contrast to most chemotherapeutic drugs, and may thus overcome drug resistance due to lacking p53 signaling [60]. While the CD95/Fas ligand and TNF-α appear as problematic for systemic treatment due to severe side effects, such as liver toxicity and induced inflammation, TRAIL has been shown to induce apoptosis in several cancers, while normal cells are largely protected [61,62,63]. In fact, TRAIL showed only few side effects in clinical trials. However, the clinical efficacy of TRAIL monotherapy was also limited [64,65,66,67]. 

## 6. Inducible Resistance Limits TRAIL-Induced Apoptosis in Melanoma Cells

TRAIL may induce apoptosis via its two agonistic receptors, DR4/TRAIL-R1 and DR5/TRAIL-R2 (Figure 1). Although melanoma cells reveal constitutive expression of DR5, this does not guarantee TRAIL sensitivity. Thus, about half of melanoma cell lines with sole DR5 expression showed intrinsic TRAIL resistance. On the other hand, all melanoma cell lines, which express DR4 in addition to DR5, were characterized by initial TRAIL sensitivity. Importantly, most melanoma tissues do express both receptors suggesting that initial TRAIL sensitivity is also characteristic for clinical melanoma [16,28].

The causes of intrinsic TRAIL resistance are not entirely understood and may also differ. Thus, reduced expression of agonistic receptors was reported for TRAIL-resistant cells of small cell lung carcinoma [68], while TRAIL-resistant cutaneous T-cell lymphoma cells were characterized by constitutive expression of c-FLIP as well as by loss of caspase-10 and Bid [69]. In other cancers, such as in cervical cancer, TRAIL resistance was related to the expression of TRAIL decoy receptors [70]. In melanoma cells, intrinsic TRAIL resistance was correlated with the expression of caspase antagonists such as elevated expression of XIAP, survivin, and c-FLIP [28,71].

Apart from the problem of possible intrinsic TRAIL resistance, initially sensitive cancer cells may develop an inducible resistance upon TRAIL treatment, as reported for several cell types such as those of breast, colon, liver, and ovarian cancer [72,73,74]. A comparable situation is seen in melanoma; namely, both DR4^+^DR5^+^ and DR4^−^DR5^+^ melanoma cells may develop inducible TRAIL resistance upon TRAIL treatment. Due to the high rate of DR4 expression in melanomas and initial TRAIL sensitivity of DR4^+^ melanoma cells, inducible TRAIL resistance appears as the major problem in melanoma. It correlates with downregulation of the two agonistic TRAIL receptors, of initiator caspases-8 and -10, as well as of some proapoptotic Bcl-2 proteins, as shown for Bax, Bid, and Bim [17,75]. While intrinsic TRAIL resistance may be excluded in patients by a forehanded screening, the development of inducible TRAIL resistance may critically limit the achievement of TRAIL-based therapy. Inducible resistance may therefore explain the so far only limited efficacy of TRAIL or TRAIL receptor agonists in clinical trials [64,65,66]. 

## 7. Multiple Strategies Sensitize Melanoma Cells for TRAIL-Induced Apoptosis

To overcome the problem of inducible TRAIL resistance, different combination strategies have been tested in tumor models [76]. With regard to melanoma cells, several distinct strategies were identified that could sensitize TRAIL-induced apoptosis. These included chemotherapeutics, irradiation, endoplasmatic reticulum (ER) stress induction, natural compounds, HDAC (histone deacetylase) inhibitors, metabolic inhibitors and signaling inhibitors, reviewed in [27], as well as inhibition of TAK1 (transforming growth factor β-activated kinase 1) [77] and interferon-β [78]. In addition, survival pathway inhibitors, presently considered for the clinic, resulted in enhanced TRAIL-induced apoptosis and were able to overcome induced TRAIL resistance, including inhibitors for BRAF and MEK [13,79], PI3K/AKT [59], ABL [80], ATM [81], PKC [82], and IKK [83].

The puzzling multitude of several, largely unrelated strategies demanded the unravelling of common principles. In melanoma cells, several lines of evidence indicated that enhanced TRAIL sensitivity goes hand in hand with cell cycle inhibition, as induced by the different strategies. Thus, TRAIL-induced apoptosis was also enhanced, and inducible resistance was overcome by culturing melanoma cells at high cell confluence or changing to serum-free conditions, which both resulted in cell cycle inhibition. The common regulation step of TRAIL sensitization in melanoma thus appears to be in parallel with the regulation of the cell cycle [84]. A cascade of kinases is involved here, in particular enclosing cyclin-dependent kinases (CDKs) [85].

## 8. Decisive Function of the SMAC/XIAP Rheostat

Several experimental data showed that the extrinsic caspase cascade in response to TRAIL was blocked in resistant melanoma cells at the level of caspase-3. Thus, the initial processing step of procaspase-3 to a 20 kDa intermediate product was seen, but its final processing to a mature 17 kDa product was prevented [13,19,83,86]. Although the initial cleavage is mediated by caspase-8, the final processing depends on caspase-3 autocatalytic activity [87]. These findings strongly indicated the role of cIAPs, which block effector caspase activity (Figure 3). 

In melanoma cells, XIAP seems to play this particular role in TRAIL sensitization. Thus, concomitant downregulation of XIAP was seen in combinations of TRAIL with ultraviolet B (UVB) radiation, HDAC inhibitors, chemotherapeutics, metabolism, and kinase inhibitors. Downregulation of XIAP further correlated with complete caspase-3 processing and enhanced apoptosis. Finally, overexpression of XIAP protected melanoma cells from apoptosis induced by TRAIL and combinations, whereas its siRNA-mediated knockdown could sensitize melanoma cells for TRAIL [19,27,83,88,89].

In apoptosis pathways, Smac serves as an antagonist of XIAP (Figure 3), suggesting that the release of Smac is a critical step in TRAIL sensitization. The important role of the Smac/XIAP rheostat was demonstrated in pancreatic and bladder cancer by using small molecule inhibitors for XIAP as well as Smac mimetics, which both enhanced TRAIL sensitivity [90,91]. In melanoma cells, the critical role of Smac was proven by knockdown strategies. Thus, Smac knockdown abolished apoptosis induced by TRAIL and combinations [13,19,83]. These data suggest the central role of the Smac/XIAP rheostat in melanoma for TRAIL sensitivity. A reinforced mitochondrial release of Smac, which is supported by pathway inhibitors, can overcome inducible TRAIL resistance in melanoma.

## 9. Critical Contribution of Mitochondrial Pathways and Bcl-2 Proteins

The critical importance of intrinsic apoptosis pathways for melanoma was already shown previously [15,36]. Thus, activation of mitochondrial pathways was a central issue for different strategies used in TRAIL sensitization, as seen by the loss of mitochondrial membrane potential and release of proapoptotic mitochondrial factors [13,27,83,84]. Importantly, apoptosis induction by the different pathway inhibitors alone was mostly insufficient, but apoptosis was efficiently induced by a combination with TRAIL. This suggests that the pathway inhibitors opened a gate towards sensitivity, but TRAIL was needed to provide the final proapoptotic signal.

In cells with induced resistance, clear signs of TRAIL signaling were still seen, such as processing of caspase-8 and of Bid [13,83]. Bid may antagonize Bcl-2 [43]; however, this was apparently not sufficient for apoptosis induction in resistant melanoma cells. Nevertheless, Bid appeared as essential for apoptosis induction by TRAIL in combination with pathway inhibitors, as shown by Bid knockdown. The critical role of Bcl-2 proteins in the enhancement of TRAIL-induced apoptosis was further demonstrated by Bax knockdown and Bcl-2 overexpression [19,59,83]. Thus, in melanoma cells with induced resistance, the initial steps of the pathway were still active; this was, however, not sufficient for apoptosis induction.

The release of Smac is tightly controlled by pro- and antiapoptotic Bcl-2 proteins (Figure 3). The principle dependency of TRAIL combinations on Bcl-2 proteins was proven by Bcl-2 overexpression and by Bax knockdown, which both diminished apoptosis induction [19,59,83,88,92]. Furthermore, downregulation of antiapoptotic Bcl-2 proteins (Bcl-2, Mcl-1, and Bcl-x_L_) was reported in melanoma cells upon treatment with different therapeutic strategies used for TRAIL sensitization, such as chemotherapeutics and inhibitors for metabolism, HDACs, and kinases. On the other hand, proapoptotic BH3-only proteins were frequently upregulated in the course of TRAIL sensitization [27]. Particularly important roles are attributed to Bim and Puma, as both may interact with all antiapoptotic Bcl-2 family members [49]. Bim or Puma were also upregulated in the course of TRAIL sensitization by HDAC, BRAF, and cell cycle inhibition [12,83,84,93]. The control of Bim by MAP kinases is based on its phosphorylation through ERK, which triggers its proteasomal degradation [11,94]. In addition, Puma expression may be upregulated in the course of MAPK inhibition through the transcription factor FoxO3a [95]. Of the quite large number of BH3-only proteins, usually only a few have been investigated in these studies. They may, therefore, have an even higher impact on TRAIL sensitization in melanoma than presently known.

The two multidomain, proapoptotic Bcl-2-related proteins Bax and Bak represent a bottleneck in mitochondrial apoptosis pathways. They are antagonized due to heterodimerization by the different antiapoptotic Bcl-2 proteins and may be activated by BH3-only proteins, either directly or indirectly through the inhibition of antiapoptotic Bcl-2 proteins (Figure 3). Both Bax and Bak can mediate apoptosis in melanoma cells [40,41], but for TRAIL-induced apoptosis and for TRAIL sensitization, particularly Bax appeared to be responsible [96]. Thus, typical activation steps for Bax (mitochondrial translocation and conformational changes) were observed in melanoma cells by the combination of TRAIL with pathway inhibitors [13,19,59,79,80,83,93]. It is worth noting that Bax activation happened early after treatment (<4 h) and was thus not a consequence of induced apoptosis. In agreement with its critical role, siRNA-mediated Bax knockdown abrogated apoptosis induction in melanoma cells by combinations of TRAIL and inhibitors [59,83]. 

Concerning the regulation of Bax, an inactivating phosphorylation at Ser-184 as well as an activating phosphorylation at Thr-167 have been reported [97,98]. By using Bax phosphorylation-specific antibodies and flow cytometry, we could prove that Bax activation by IKK, PI3K, and AKT inhibitors correlated with suppressed Ser-184 phosphorylation as well as with enhanced Thr-167 phosphorylation. Both changes appeared as immediate effects (within 1–2 h) and were independent of induced apoptosis [59,83]. The inhibited phosphorylation at Ser-184 had been related to AKT activity [45,97], whereas the enhanced phosphorylation at Thr-167 was related to JNK, p38, and ERK pathways [98,99]. With regard to the Thr-167 phosphorylation in the course of PI3K inhibition, we have seen a further relation to the production of reactive oxygen species (ROS). Thus, antioxidants prevented Thr-167 phosphorylation and partially rescued melanoma cells from wortmannin/TRAIL-induced apoptosis [59]. The significance of ROS for apoptosis regulation in melanoma cells was reported [100,101,102], and ROS was also involved in sensitization of melanoma cells for TRAIL by cell cycle inhibition [84]. Together, these data suggest that an early Bax activation is the critical step in sensitization of melanoma cells for TRAIL, which then allows release of Smac.

## 10. Conclusions

TNF-related apoptosis-inducing ligand (TRAIL) represents a promising antitumor strategy, which may also apply for the treatment of cutaneous melanoma. A particular task is to overcome the problem of inducible TRAIL resistance. Several strategies for enhancing TRAIL-induced apoptosis and for overcoming inducible TRAIL resistance are presently evaluated. One important approach is the development of novel TRAIL receptor agonists, as the first-generation TRAIL receptor agonists did not show sufficient clinical efficacy. Thus, hvTRA (APG350) is a synthetic fusion protein which consists of two covalently linked, trivalent single-chain TRAIL receptor-binding domains, resulting in a hexavalent binding mode, which can strongly enhance the proapoptotic signal [103]. In melanoma, hvTRA efficiently induced apoptosis and led to sustained growth reduction in cell lines and xenograft models [104]. 

Several lines of evidence have underlined the critical role of the Smac/XIAP rheostat in induced TRAIL resistance in melanoma cells. Smac mimetics are presently evaluated for cancer therapy [105]. Although melanoma cells are largely insensitive to these mimetic drugs when used as single agents, combinations with TRAIL or TRAIL receptor agonists appear as promising [106]. Thus, the IAP antagonists Birinapant and AT-406 could sensitize BRAFV600E colorectal tumor cells for TRAIL-induced apoptosis [107].

As TRAIL-induced apoptosis in melanoma cells and Smac release are essentially controlled by Bcl-2 proteins, the targeting of antiapoptotic Bcl-2 proteins appears as an important strategy for enhancing TRAIL sensitivity. BH3-only proteins function as sensitizers for apoptosis induction, and particular roles for apoptosis regulation in melanoma have been attributed to Bim and Puma [38,48,49]. A number of BH3 mimetics have been established, e.g., ABT-737, ABT-263, A-1155463, and S63845, as well as ABT-199 (Venetoclax), which has been approved for treatment of refractory chronic lymphocytic leukemia [38,108,109]. Although BH3 mimetics were less effective in melanoma cells as a single therapy, they revealed synergistic effects in combinations, e.g., with immunotoxins or the proteasome inhibitor bortezomib [42,110]. Due to the in vitro data, one might also expect good combination effects for BH3 mimetics and TRAIL in melanoma cells, as has been previously shown in glioma cells [111].

**Figure 4:**
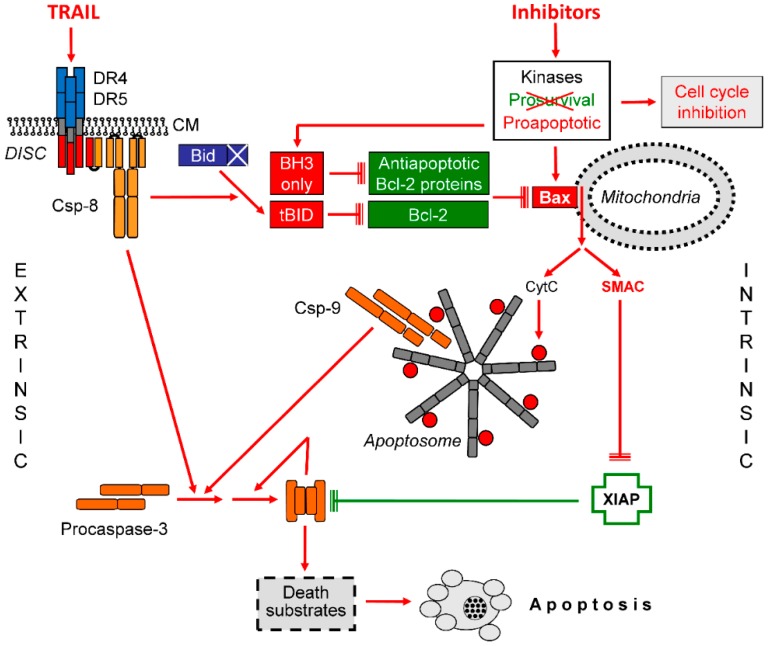
Steps of TNF-related apoptosis-inducing ligand (TRAIL) sensitization in melanoma. The interplay of extrinsic and intrinsic apoptosis pathways for TRAIL signaling in melanoma cells is shown. Important steps are the activation of Bax and the release of Smac. Further explanations are given in the text. Abbreviations: CM, cytoplasma membrane; DISC, death-inducing signaling complex.

A major issue of this review article is the consideration of combinations of pathway inhibitors and TRAIL for melanoma therapy. An important advantage is that many kinase inhibitors are already approved for the clinic. The mechanism of enhanced TRAIL-induced apoptosis by pathway inhibitors is based on the understanding that despite induced resistance, TRAIL still results in caspase-8 and Bid activation. Bid can antagonize Bcl-2, which is, however, not sufficient for activation of the mitochondrial apoptosis pathway. This may depend on the activity of other antiapoptotic Bcl-2 proteins and/or on an inactivated Bax, as regulated by kinases (Figure 4). 

Upon TRAIL sensitization by pathway inhibitors, the regulation level of kinases is decisively changed, resulting in cell cycle arrest as well as in Bax activation. The regulation of Bax may further depend on BH3-only proteins. Now, the mitochondrial gate is open, Smac is released, and the inhibition of apoptosis by XIAP is overcome. Then, caspase-3 can undergo the final activation step through autoprocessing, and apoptosis is induced (Figure 4). With an improved understanding of the interplay of the pathway inhibitors and TRAIL, new combination strategies may be designed, which may allow the utilization of the proapoptotic potential of TRAIL for melanoma therapy.
